# Changes in the Sexual Behavior of Partners in Each Trimester of Pregnancy in Otwock in Polish Couples

**DOI:** 10.3390/ijerph19052921

**Published:** 2022-03-02

**Authors:** Robert Kulhawik, Katarzyna Zborowska, Beniamin Oskar Grabarek, Dariusz Boroń, Violetta Skrzypulec-Plinta, Agnieszka Drosdzol-Cop

**Affiliations:** 1 Sexology and Pathology Clinic, 02-001 Warsaw, Poland; 2 Department of Reproductive Health and Sexology, Department of Woman’s Health, School of Health Sciences in Katowice, Medical University of Silesia in Katowice, 40-555 Katowice, Poland; kzborowska@sum.edu.pl (K.Z.); vskrzypulec@sum.edu.pl (V.S.-P.); 3 Department of Histology, Cytophysiology, and Embryology in Zabrze, Faculty of Medicine in Zabrze, University of Technology in Katowice, Academia of Silesia, 41-800 Zabrze, Poland; bgrabarek7@gmail.com (B.O.G.); dariusz@boron.pl (D.B.); 4 Department of Gynecology and Obstetrics in Zabrze, Faculty of Medicine in Zabrze, University of Technology in Katowice, Academia of Silesia, 41-800 Zabrze, Poland; 5 Department of Gynecology and Obstetrics with Gynecologic Oncology, Ludwik Rydygier Memorial Specialized Hospital, 31-826 Kraków, Poland; 6 Chair and Department of Gynecology, Obstetrics and Gynecological Oncology, Medical University of Silesia in Katowice, 40-472 Katowice, Poland; cor111@poczta.onet.pl

**Keywords:** sexual intercourse, sexuality, pregnancy, couples, Polish observational study

## Abstract

The aim of this study was to improve knowledge regarding pregnant couples by assessing changes in their sexual behavior, the reasons for the frequency and forms of intercourse, and the self-sexuality of partners in each trimester of pregnancy. As a result, 100 couples were qualified to take part in the final study, with 100 men and 100 women examined at intervals equivalent to the trimesters of pregnancy. These women (*n* = 100) and men (*n* = 100) were partners. Each of the studied couples was assessed in the trimesters of pregnancy. A prospective examination was conducted based on the observation of the same people, which were heterosexual couples, throughout pregnancy. The data used in the study was obtained using Davies’ Sexual Satisfaction Scale (DSSS), as well as an original survey on the sexuality of people during pregnancy (SARSS). The survey allowed us to obtain sociodemographic (metrics) information along with information regarding the sexual activity, satisfaction, and sexual attraction of the partners. All questionnaire questions were answered in paper form. There was a statistically significant drop in sexual intercourse from the first trimester in the second and third trimesters. The frequency of masturbation increased in men and decreased in women. The most common cause of sexual abstinence during the pregnancy was cited as fear for the fetus by all genders. A statistical analysis indicated that the average overall scores of the DSSS for the examined women in the first trimester of pregnancy were statistically significantly higher than in the two subsequent trimesters (*p* < 0.05). Sexual satisfaction, measured through both DSSS and SARSS, was also strongly correlated with the level of satisfaction in the assessment of the partner relationship and with the sense of one’s own attractiveness (*p* < 0.05). Changes in sexual behavior and sexual problems are often exposed or worsened during a first pregnancy and can have negative impacts on a person and the future of relationships. Medical staff should be trained in the assessment of sexual difficulties in people during pregnancy, in order to conduct reliable education and increase the awareness of couples regarding sexual and reproductive health.

## 1. Introduction

Research regarding the sexuality of women, including that of pregnant women, has recently grown in popularity. The World Health Organization (WHO) defines sexual health as “a state of physical, emotional, mental and social well-being related to sexuality; it is not merely the absence of disease, dysfunction or infirmity” [1]. During pregnancy, a gradual decline in sexual activity, interest and satisfaction is observed. This is related to changes in sexual life, body image, and the neurological and hormonal systems, along with disturbances in psychological and emotional nature [2,3]. Throughout pregnancy, significant, deep physical and emotional changes occur among pregnant women and their partners. The majority of couples remain sexually active during pregnancy; however, some pregnant women are unsure of the safety of sexual activity and the effect on the well-being of the fetus, as well as the maintenance of the pregnancy [4]. Conducted observations also indicate that the sexual activity of couples decreases with the successive trimesters of pregnancy [5,6,7].

As early as 1966, Masters and Johnson stated that, in the first trimester of pregnancy, pregnant women’s sexual interest clearly decreased [8]. The main cause was the physiological symptoms occurring in the first trimester of pregnancy, such as sleepiness, breast pain, nausea, vomiting, and mood disorders. There are also concerns about damaging the embryo or miscarriage due to sexual contact [9,10,11,12].

On the other hand, the first trimester brings changes with it that actually favor sexual intercourse. Most of all, the couple no longer needs to worry about contraception and protection from unwanted pregnancy [13]; this certainly leads to relaxation.

In the second trimester of pregnancy, there is a visible increase in the number of sexual encounters for the vast majority of women; additionally, there is an enrichment of sexual experience, as well as an increase in fantasies and erotic dreams, regardless of the number of previous pregnancies [14]. This is partly caused by changes of a physical nature, such as the increased congestion of the genital organs and more intense lubrication of the vaginal walls resulting from hormones; however, it is also partly psychological acclimation to the state of pregnancy and the acceptance of one’s own appearance. A woman’s greater openness and willingness to talk about sexual fantasies initiates the return of old activity, often in an even more intimate version, allowing for the rediscovery of sexual satisfaction [2,3,15].

In the third trimester of pregnancy, there is a marked decrease in sexual activity, a decrease in the frequency of intercourse and impairment of the ability to experience orgasm in both nulliparous and multiparous women. Undoubtedly, according to Eisenberg et al. [16], this is a consequence of fear for the child or fear of premature labor—after even half an hour of sexual intercourse, uterine contractions may occur—but they are not labor contractions [16,17]. The average length of intercourse among Poles has not changed since 2005 and is still between 13 and 15 min. On the other hand, the time of foreplay has shrunk to 15 min, which is 5 min lower than in previous years [18].

Ilska et al., conducting research on a group of Polish pregnant women with normal and high-risk pregnancies, found that concerns about sexual activity were reported by almost half of the respondents from the normal pregnancy group (47.0%), while in the group of pregnant women at risk, the percentage was still higher (62.7%). The most frequently reported concerns, especially among high-risk pregnant women, are: fear of miscarriage/preterm labor (34.9%); fear of “harming the baby” (22.2%); fear of infection (22.2%) [19].

In the final weeks of pregnancy, there is a justifiable need to discontinue sexual intercourse due to risk of infection. It is an absolute necessity for a pregnant woman to abstain from intercourse in case of some complications in the course of pregnancy, such as premature drainage of amniotic fluid and preterm labor. The sexual activity of women therefore resembles a sinusoidal curve. The individual trimesters of pregnancy tend to dictate the quality and quantity of intercourse between partners [20].

A dyadic study of behavior is where the study group consists of two related people, such as married couples, roommates, friends, or coworkers [21,22]. Ramsdell et al. [23] conducted research on various elements of intimate relationships and overall satisfaction with the relationship, separately in pregnant women, and their male partners. These authors observed that emotional intimacy, support, and respect were more important for women than for men. Furthermore, they determined that, for example, the level of emotional intimacy, closeness, and conflict resolution had a larger influence on the satisfaction from the relationship, than, for example, the quality of sexual relations [23].

Of course, it should be remembered that not just heterosexual people, or people whose psychological gender is the same as their biological sex, can plan to be and be pregnant. People who do not identify as women, or who are trans, nonbinary or otherwise gender diverse can also be pregnant [24,25,26].

The aim of this study was to improve knowledge regarding pregnant couples by assessing changes in their sexual behavior, the reasons for the frequency and forms of intercourse and the self-sexuality of partners in each trimester of pregnancy.

## 2. Materials and Methods

### 2.1. General Procedures

A prospective examination was conducted based on the observation of the same people, which were heterosexual couples, throughout pregnancy. The data used in the study were obtained using Davies’ Sexual Satisfaction Scale (DSSS), as well as an original survey on the sexuality of people during pregnancy (SARSS). The survey allowed us to obtain sociodemographic (metrics) information along with information regarding the sexual activity, satisfaction, and sexual attraction of the partners. All questions were answered in the questionnaires in paper form.

### 2.2. Participants

The study was conducted among people living in the Otwock county of the Mazovian voivodeship at a medical center, with consent to conduct the research obtained from the Gynecology and Obstetrics Clinic of the Family Health Center (05-400 Otwock, ul. Grunwaldzka 13, Poland). The recruitment of patients and the entire study took place in the Gynecological Outpatient Clinic. The patients were not hospitalized. The study was conducted in the period from 2 January 2018 to 20 March 2019.

The number of inhabitants of Otwock, according to data of the Central Statistical Office of 2017, was 44,635. The number of participants in the study was determined using the statistical tool available at https://www.naukowiec.org/dobor.html (accessed on 10 October 2017). For a population of 44,365 inhabitants, the maximum error value was estimated at 7%. Therefore, assuming a *p*-value < 0.05, the required number of respondents in the study is 195. In our study, it is 200 people [27]. Table 1 presents the inclusion and exclusion criteria.

### 2.3. Recruitment Criteria

Couples whose pregnancies were conducted at the Gynecology and Obstetrics Clinic of the Family Health Center in Otwock participated in the study. The doctor introduced the couples to the possibility of participating in the study, acquainted them with the study protocol, and asked for their consent to participate in the study. If a couple decided to participate in the study, they became its member. If the decision was made not to participate in the study, there were no negative consequences for the couple. Their pregnancy was conducted in accordance with the applicable rules.

Out of 124 couples in the study, 16 withdrew from the study because they found the discussion of sexuality inconsistent with their conscience and religion. The remaining couples were excluded due to complications during pregnancy: preterm labor at 32 weeks of gestation (*n* = 2); or a significant lack of responses (*n* = 6). There was no incentive for participation.

To conduct the study correctly, each of the examined couples was randomly assigned an examination number and given a set of questionnaires for both men and women. The code representing the examination set was determined using the formula:

- for men: x/y/M
- for women: x/y/K

where x is a random one-time number representing the ordinal number of the tested pairs between 1 and 100, with the same set of questionnaires being used for all respondents; y represents the duration of pregnancy split into trimesters: I (8–12 weeks); II (20–24 weeks); III (32–36 weeks). The division into specific weeks during a given trimester has been defined so that when filling in the questionnaire, information relevant to a given trimester is collected.

Before starting the study, each pair was informed about the purpose and course of the study in order to obtain informed consent to participate in the study. Then, instructions on how to fill in the questionnaire were provided.

### 2.4. Collection Variables

Metrics included data about the study participants, such as sex, age, place of residence, education, marital status, and professional situation. Each question, except the one about age, was multiple choice. Additionally, questions were created about the number of previous pregnancies, any complications during the current pregnancy, the fertilization method, and any infertility treatment. The obtained answers allowed us to conduct a statistical analysis of the data obtained only from the couples who were qualified to participate in the study, as specified in the inclusion and exclusion criteria above.

### 2.5. The Self-Assessment of the Respondents Sexual Satisfaction Scale (SARSS) and Davies’ Sexual Satisfaction Scale (DSSS)

Sexual satisfaction was assessed in two ways: through a 10-point scale and the standardized DSSS scale during the trimesters of the pregnancy.

The self-assessment of the respondents in terms of sexual satisfaction was determined based on a 10-point scale of answers (1–2—definitely not; 3–4—probably not; 5–7—rather yes; 8–10—definitely yes). The respondents had to answer the question, “Are you currently sexually satisfied? (please specify on a scale of 1–10)”. For the self-assessment of the respondents, in terms of sexual satisfaction, Cronbach’s Alpha indicated a high consistency, 0.93.

The level of sexual satisfaction was measured using Davies’ Sexual Satisfaction Scale (DSSS), translated into Polish by Szumski and Małecki. The scale is comprised of 21 statements, which the participant answered using a five-point Likert scale (1—Strongly Disagree; 2—Disagree; 3—Undecided; 4—Agree; 5—Strongly Agree). The score obtained from the examined individual was in the range of 21–105. For the Sexual Satisfaction Scale, Cronbach’s Alpha indicated a high consistency, 0.83.

The questionnaire comprised three subsections:

- Physical sexual satisfaction—relating to the assessment of the quality of sexual contact in a relationship, the sexual abilities of the partner, and the satisfaction of their own sexual needs in a relationship. The score obtained from the examined individual was in the range of 11 to 55;
- Emotional sexual satisfaction—measures the affective feelings towards sex and the partner’s behavior as well as the feelings towards their partner. The score obtained from the examined individual was in the range of 4 to 20;
- Control-related sexual satisfaction—relates to the assessment of their own influence over how, when and if at all the person has sexual contact. The score obtained from the examined individual was in the range of 6 to 30.

### 2.6. Statistical Analysis

Statistical analysis and visualization of the obtained results was conducted using Microsoft Excel (Office 365) and IBM SPSS Statistic Version 21 (Cracow, Poland).

The Shapiro–Wilk test was used to analyze the normal distribution of the obtained data. Due to the fact that the test confirmed a normal distribution (*p* > 0.05), parameter tests were used in the statistical analysis of our study. Data was presented as an average with standard deviation. Furthermore, Student’s *t*-test was used to analyze differences between the responses provided by men and women. When there were more than two groups, an ANOVA variance analysis was performed, followed by Tukey’s post hoc test. The analysis of the differences in the numbers between the variables expressed on the nominal scale was performed using the chi-squared test. Pearson’s correlation (r) analysis was used to determine the existence of correlations between features.

In all the tests used, results with a significance level of *p* < 0.05 were assumed to be statistically significant.

### 2.7. Ethics

This study was conducted according to the guidelines of the Declaration of Helsinki, and approval was obtained from the Bioethical Committee operating at the Medical University of Silesia in Katowice, Poland, no. KNW/0022/KB/280/17 (date: 12 December 2017).

Each of the couples were informed of the purpose and duration of this study in order to obtain informed consent. Next, the participants were instructed on how to correctly answer the questionnaires. Each of the participants were also informed of the confidentiality of this study, as well as their anonymity and voluntary participation as well as the ability to withdraw from completing the questionnaires. Only the first author of the publication had access to nonanonymized research. After they were anonymized, the rest of the research team had access to the results. The data was stored in accordance with applicable laws.

## 3. Results

### 3.1. Results of the Characteristics of Women, Men and Couples Participating in the Study

**Figure 1:**
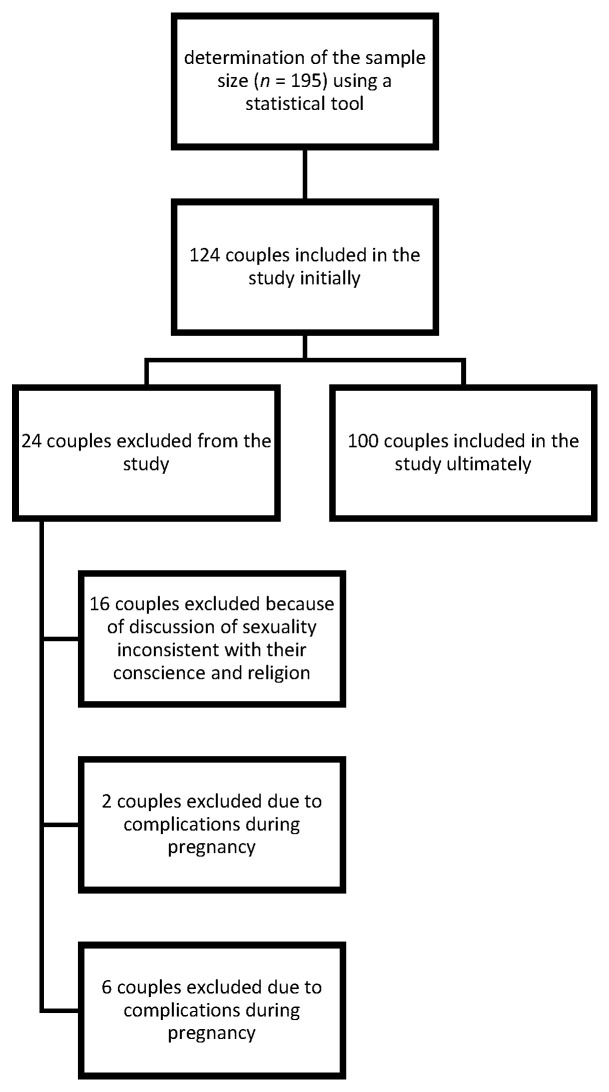
Sample selection process.

As a result, 100 couples qualified to take part in the final study, with 100 men and 100 women examined at intervals equivalent to the trimesters of pregnancy. These women (*n* = 100) and men (*n* = 100) were partners (Figure 1). Each of the studied couples was assessed in the trimesters of pregnancy. The age of the participants was between 20–46 years old (33.45 ± 5.60). The average age of the examined women was 31.76 ± 4.60, while for men it was 35.14 ± 5.75. Each of the participants responded separately to eliminate the influence their partner’s presence may have had.

The majority of the participants were inhabitants of an urban agglomeration of up to 100,000 residents (77%; *n* = 154), while the remainder defined their place of residence as a city with between 100,000 and 499,000 inhabitants (14%; *n* = 28) or as a village (9%; *n* = 18). The participant were residents of Otwock county, which encompasses five towns—Otwock, Józefów, Karczew, Celestynów and Wiązowna—with an additional 135 villages also being located in the county; altogether, the county has an estimated total population of 123,000 people. The study group was dominated by people who had completed higher education (68%); when split between the sexes, 76% of the examined women (*n* = 76) and 60% of the men (*n* = 60) had completed higher education. The examined couples were generally characterized by both partners possessing an equal level of education, with 60% (*n* = 60 couples) of the couples having a higher education, whereas the remaining couples had secondary education (22%; *n* = 22 couples) or a mixture of secondary and higher education (18%; *n* = 18 couples). The participants were mainly white-collar workers (*n* = 116), of which most white-collar workers were women (*n* = 76), while only 16% of the participants did not work at all (10% of women (*n* = 10) and 6% of men (*n* = 6)). The participants were also asked about their economic situation using a 10-point scale. The average score for their economic situation was 6.71 ± 1.73; when split based on sex, it was 6.74 ± 1.56 for women and 6.68 ± 1.9 for men. The economic situation of the respondents could hence be described as moderately satisfactory. No statistically significant differences between the sexes in terms of education, profession or economic situation were indicated (*p* > 0.05; post hoc Tukey’s test). The surveyed couples were also asked to describe their relationship status. The vast majority of the respondents declared that they were married (66%; *n* = 66 couples); the remainder identified their relationship as a partnership (34%; *n* = 34 couples). The mean duration of the relationship for the studied couples was 3.8 ± 0.14 years, the duration of which was statistically significantly described as longer by men than by women (Student’s *t*-test; *p* = 0.02). Detailed data is presented in Table 2. The men participating in the study defined their gender identity as: male gender—49; female sex—1; undefined gender—0; transvestite sex—0; transgender gender—0; transsexual sex—0; autogynephilic sex—0 (*p* > 0.05). In turn, women defined their gender as: male gender—0; female sex—50; unspecified gender—0; transvestite sex—0; transgender gender—0; transsexual sex—0; autogynephilic sex—0 (*p* > 0.05). The study group was not asked about faith because the vast majority of the population of this county are practicing Catholics, and there were no statistically significant relationships between the characteristics of sexual activity and sociodemographic data such as age, place of residence, professional situation, etc.

### 3.2. Evaluation of Relationship with the Current Partner and Assessment of Own and Partner’s Physical Attractiveness

During pregnancy, the respondents were asked to evaluate their relationship with their current partner and to assess their own and their partner’s physical attractiveness. Most of the respondents were satisfied with their current relationship with their partner (8.74 ± 0.137). Statistically significant differences were not found between the assessment of the relationship with the current partner for the respondents (*p* = 0.92). However, the assessment of one’s own physical attractiveness differed between men and women, with the average self-assessment of attractiveness for women being 4.88 ± 1.37, while for men, it was 7.2 ± 0.14 (*p* = 0.00). Men self-assessed their physical attractiveness statistically significantly higher compared to women. In the case of assessing the physical attractiveness of their partner, both men and women rated the attractiveness of their partners highly, with the minimum value being 7 and the maximum being 10; the average for women was 8.7 ± 0.160, and for men, it was 9.4 ± 0.11 (*p* = 0.76; Table 3).

### 3.3. Characteristics of Partnerships and Sexual Activity during Pregnancy

**Figure 2:**
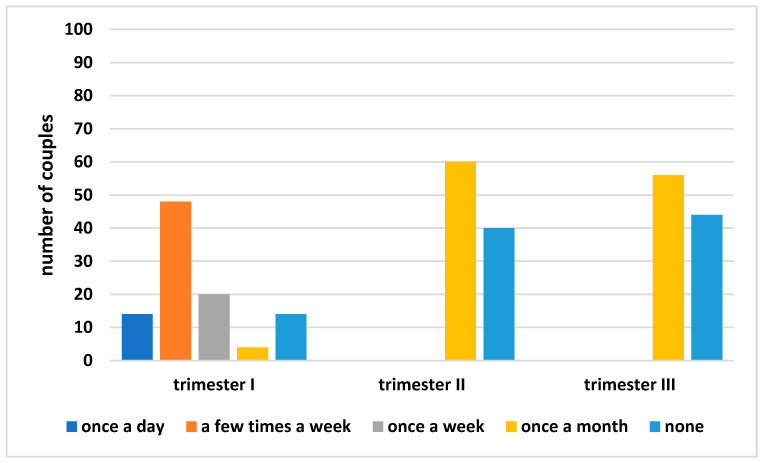
The frequency of sexual intercourse of the couples depending on the pregnancy trimester.

The participant was asked whether they had sexual intercourse, as well as the frequency of sexual intercourse during pregnancy. Sexual intercourse in the first trimester was reported by 86% (*n* = 86 couples) of the respondents, who most often described the frequency as “few times a week (more than 3 times a week)” (48%; *n* = 48 couples) or “once a week” (20%; *n* = 20 couples); the remaining respondents described their sexual activity as “once a day” (14%; *n* = 14 couples). In the second and third trimester, a statistically significant drop in the frequency of sexual intercourse was noted (Tukey’s post hoc test; *p* = 0.00): 60% of the participants had sexual intercourse, and the vast majority described their frequency of sexual intercourse as “once a month” (*n* = 60 couples). The third trimester of pregnancy was characterized by comparable results to the second trimester; in this trimester, sexual activity of “once a month” was declared by 56% (*n* = 56 couples), which constitutes the entire group of sexually active participants in the third trimester of pregnancy. The analysis of variance, followed by a Tukey’s post hoc test, indicated statistically significant differences in the frequency of sexual activity regardless of the trimester (*p* = 0.00). The obtained results were presented on the Figure 2.

**Figure 3:**
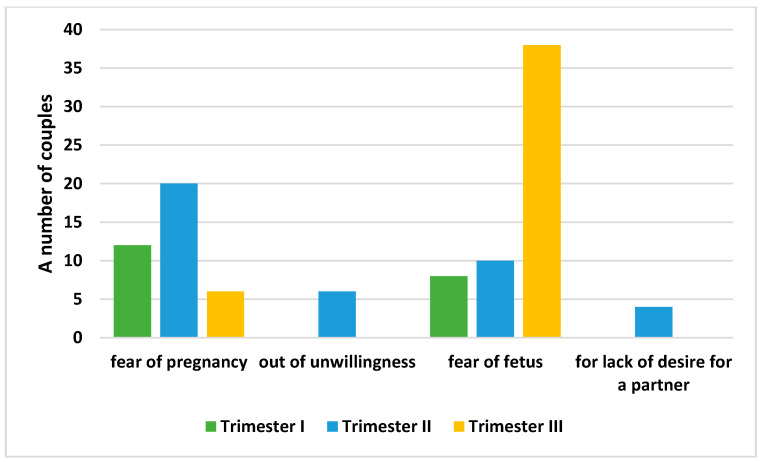
Reasons for sexual abstinence.

Couples who did not have sexual intercourse during pregnancy were asked about their reason for sexual abstinence (multiple-choice question). In the first trimester of pregnancy, a lack of sexual activity was declared by 14% of the examined couples (*n* = 14 couples), the most commonly indicated cause being fear of complications in pregnancy (maintenance of pregnancy); another cause was the absence of the partner, while other reasons indicated by women were “it’s the right thing to do” or “now is not the time for sex”. In the case of men, “fear for the fetus” was the dominant answer, as well as maintenance of the pregnancy by the partner. In the second trimester of pregnancy, for both men and women, sexual abstinence was caused by unwillingness (women), unwillingness from the partner (men) or fear for the fetus and the course of pregnancy. The answers of the couples were dominated by unanimous responses regarding concerns about pregnancy and concerns about the fetus. The final trimester was characterized by a cessation of sexual activity due to fear for the fetus in the case of men, as well as unwillingness to be sexually active from women. Women normally ceased sexual activity due to changes in outer appearance connected with the pregnancy, as well as fear for the fetus and unwillingness. The dominant, statistically significant reason for sexual abstinence of the couples was fear for the fetus (post hoc Tukey’s test; *p* = 0.00). The obtained results were presented on the Figure 3.

In connection with engaging in sexual activity, the examined couples were asked about their preferred sexual positions during pregnancy. The participants were asked to indicate their most commonly preferred sexual position during sexual intercourse throughout pregnancy. During pregnancy, the most common dominant positions among women were the knee–elbow position and on their side. In the case of men, the most often declared position was the knee–elbow position on the side and also positions in which the partner is on top (cowgirl); additionally, some of the men indicated oral–genital contact as their preferred sexual position, which was not indicated by the examined women. The conducted statistical analysis did not indicate any dominant trend regarding the selection of sexual positions during pregnancy; furthermore, no statistically significant differences in terms of changes in sexual positions during pregnancy were noted (*p* > 0.05).

The participants were also asked about their masturbatory behavior as well as the occurrence of erotic dreams during pregnancy. Half of the participants declared that they performed a masturbatory act during the first trimester of pregnancy (52%; *n* = 104). The vast majority of these people were men (76%; *n* = 76), who declared that they masturbated “once a week” (0%; *n* = 20) or “several times a week” (30%; *n* = 30), with 26 men declaring that they masturbated “daily.” Only 28% (*n* = 28) of women masturbated in the first trimester and 2% (*n* = 2) in both the second and third trimester declared their frequency as “less than once a week.” For men, the second and third trimesters were characterized by a statistically significant increase in the frequency of masturbation (*p* = 0.00; post hoc Tukey’s test; Table 3). The data indicated that all the men (*n* = 100) masturbated “daily” or “a few times a week” in the second and third trimesters. In the case of women, no statistically significant changes regarding masturbatory acts and their frequency during pregnancy were noted (*p* = 0.00; post hoc Tukey’s test). Statistically significant differences between men and women were observed regardless of the trimester (*p* = 0.00; Student’s *t*-test; Table 4).

### 3.4. The Results of Sexual Satisfaction of the Respondents during Pregnancy Based on Self-Assessment and the Davies’s Sexual Satisfaction Scale

**Figure 4:**
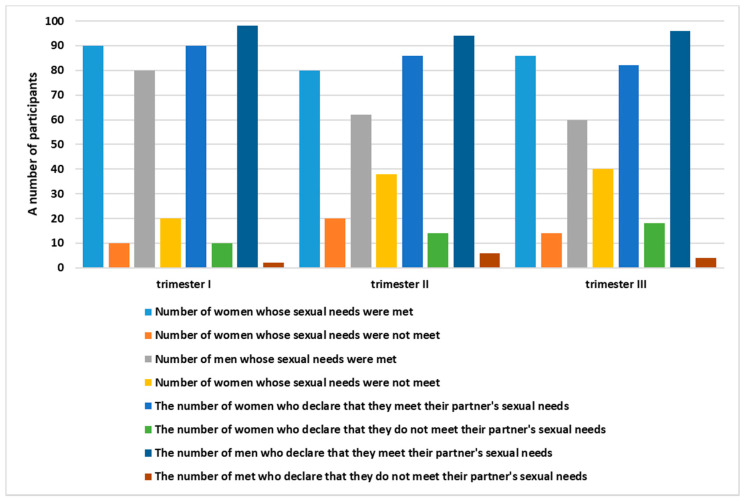
Satisfaction of sexual needs during the course of pregnancy.

In the conducted study, the respondents were asked if their sexual needs were satisfied, as well as if they themselves were satisfying the sexual needs of their partner during pregnancy. The satisfaction of one’s own sexual needs, as well as their partner’s, was declared by the vast majority of respondents. In the subsequent trimesters, the results were more varied for men. It was noticed that, for men, the percentage of respondents whose sexual needs were not satisfied increased. Interestingly, in the case of the question “Are you satisfying the sexual needs of your partner?”, it can be observed that the couples mutually satisfied each other’s sexual needs. By analyzing this question, it can, however, be inferred that the men did not express their sexual needs and the women satisfied the sexual needs of their partners in accordance with their own opinion, which did not necessarily translate to the sexual needs of the men in the group actually being satisfied (Figure 4).

**Figure 5:**
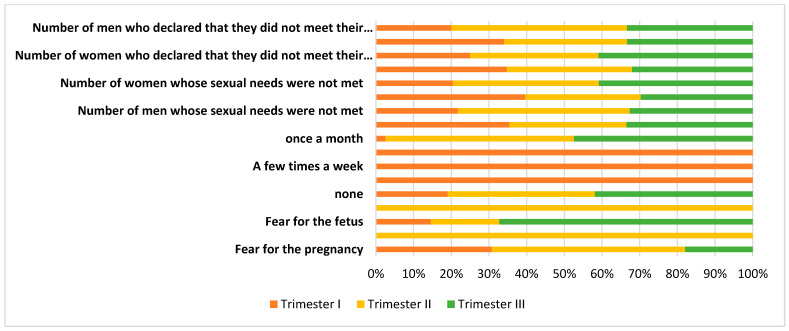
An 100% cumulative chart showing the reasons for sexual absenteeism, frequency of sexual activity and meeting the expectations of the couple in each trimester of pregnancy.

In turn, Figure 5 summarizes the information from Figure 2, Figure 3 and Figure 4.

The self-esteem of the respondents in terms of sexual satisfaction was determined using a 10-point scale of responses. The respondents in the first trimester of pregnancy described their sexual satisfaction as “satisfactory,” with an average result of 8.03 ± 2.24; for women, this was 7.24 ± 2.09, whereas for men, it was 8.82 ± 2.13. The remaining two trimesters were characterized by similar values for the entire study group. Statistically significant differences in the assessment of sexual satisfaction were noted between the trimesters in the group of women (summarized in Table 5). In both the second and third trimesters of pregnancy, women achieved statistically significantly lower results in comparison to the first trimester, as well as in comparison to men. The “satisfactory” assessment of the sexual satisfaction of women in the first trimester of pregnancy (7.24) decreased in the second trimester to 5.88 ± 2.78 and again in the third (to 5.78 ± 2.49) (Table 5).

The sexual satisfaction of the participants during pregnancy was also assessed using the sexual satisfaction scale (DSSS), which is composed of 21 diagnostic questions. The respondent was tasked to indicate, on a five-point scale, to what degree they agreed with a particular statement. The average values for women can be described as “average” (first trimester) or “low” (second and third trimester). For men, regardless of the duration of the pregnancy, they had high scores. The conducted statistical analysis indicated that statistically significant differences existed in the overall score of the sexual satisfaction scale (DSSS) for women. For men, no statistically significant differences were indicated between the trimesters of pregnancy; however, their scores were statistically significantly higher compared to the range of overall and subcategory scores of their partners (post hoc Tukey’s test *p* = 0.00).

The statistical analysis indicated that the average overall scores of the sexual satisfaction scale for the examined women in the first trimester of pregnancy were statistically significantly higher than in the two subsequent trimesters (Tukey’s post hoc test; *p* = 0.00) and also that there was a statistically significant drop in overall sexual satisfaction between the trimesters of pregnancy (Student’s *t*-test; *p* ≤ 0.00). The specific data is presented in Table 5.

The relationship between sexual satisfaction (DSSS) and sexual life self-esteem (SARSS) proved to be very strong, which means that the self-esteem of the respondents is consistent with the results obtained from the standardized questionnaire, and high self-esteem is accompanied by high scores in the subjectively assessed sexual satisfaction. Sexual satisfaction, measured through both DSSS and SARSS, was also strongly correlated with the level of satisfaction in the assessment of the partner relationship and with the sense of one’s own attractiveness. Statistically significant correlations were noted between DSSS and SARSS (for women r = 0.323; for men r = 0.436); DSSS and physical attractiveness (for women r = 0.469; for men r = 0.514); DSSS and SARSS (for women r = 0.314; for men r = 0.398); SS and assessment of partnership relations (for women r = 0.574; for men r = 0.499); SS and physical attractiveness (for women r = 0.620; for men r = 0.754).

### 3.5. Sources of Information about Sexual Contact during Pregnancy

The study group was asked questions regarding advice they had received, as well as their sources of information about sexual contact during pregnancy. The conducted analysis indicated that only 31% of the respondents consulted a specialist for advice. Most commonly, women (42%; *n* = 42) approached their gynecologist (14%; *n* = 14) or midwife (28%; *n* = 28) for advice and 16% (*n* = 16) utilized the Internet or magazines for women. In the case of men (*n* = 20), the most common sources of advice were their partner’s gynecologist (*n* = 4), as well as people close to them (friends). Most of the participants (87%; *n* = 87) used other sources of information regarding sexual contact during pregnancy, with this most often being identified as the Internet or magazines for women.

## 4. Discussion

To this day, there is debate about the influence of pregnancy on female sexuality and the influence of sexual intercourse on the course of pregnancy [28,29,30,31,32,33,34]. Unfortunately, within society, as well as among medical personnel, there are many unscientific views regarding sexual intercourse during pregnancy. There is a current belief in society, as well as among senior medics, that sex during pregnancy may threaten the well-being of the fetus, force premature birth, or is simply inappropriate [35,36]. The participants indicated that sexual abstinence during pregnancy is connected with fear for the fetus. In connection with this, the study group was asked questions regarding advice they had received and sources of information they had consulted about sexual contact during pregnancy.

Unfortunately, scientific research indicates that doctors do not provide expectant parents with information regarding the changes they can expect in the intimate sphere of their lives. If given information on how sexuality and sexual contact change during pregnancy, people can get over their fears and anxieties, and sexual intercourse may be easier to accept, making it more satisfactory. The knowledge that future parents obtain can allow them to undertake efforts to decrease the negative factors influencing sexual relations and accept those factors that cannot be changed [11].

However, considering all the evidence, often contradicting each other [35,36,37,38], sexual contact during pregnancy does not have a harmful effect on the fetus. However, care should be taken to understand appropriate body positions during pregnancy [37,38].

In the first trimester, several types of ailments occur in women, such as emotional lability, anxiety, nausea, breast pain, and sleepiness, all of which negatively affect the libido [39,40,41]. According to Lew-Starowicz et al. [42] and Skrzypulec-Plinta et al. [43], the first trimester is correlated with a lower frequency of sexual intercourse, and due to this, a decrease in sexual satisfaction, mainly due to nausea, vomiting, and breast hypersensitivity as well as a general worsening in well-being. The modification of the position of the partners’ bodies, with the woman lying on her side with her back to the man, tilted away from him and with bent knees, regulates the depth of insertion; the more she is tilted, the deeper the penetration. Slow undulation and no pressure on the abdomen allow the partners to enjoy the intercourse. This arrangement allows both partners to be active and is suitable for the entire period of pregnancy if there are no contraindications. This situation can be different in the second trimester of pregnancy. Women may be more willing to engage in sexual activity as the blood supply to the pelvic area increases, which significantly influences the ease of having sexual intercourse and achieving sexual satisfaction [44]. The second trimester normally allows for any positional modifications during sexual intercourse, under the condition that the stomach is not squeezed. Sexual contact can be emotionally problematic for men due to the feeling of a “third person” being there during sexual contact, but this opinion was not seen in our analysis. Moreover, feeling the movement of the fetus during the second trimester influences the quality of sexual contact and the choice of sexual positions [40]. The situation changes again in the most difficult third trimester of pregnancy. Traditional sexual functions become more difficult and rarer, resulting from physical pains and obstacles, such as diminished desire, lubrication, and sexual satisfaction [45]. The woman feels less attractive as a sexual partner due to growing fatigue, shortness of breath, swelling, cramps, and general physical exhaustion [46,47,48]. Lack of desire often results in insufficient sexual arousal, which is connected with lower lubrication of the vagina, lowered satisfaction, and a lack of orgasm [46,48].

The results of our research on this subject coincide with the studies conducted by Makara-Studzińska et al. [49] and Sipiński et al. [50], who discovered that, with the progression of pregnancy, sexual activity decreases [49,50]. Commonly, it is believed that female sexual activity during pregnancy depends on the cultural traditions of the society the women live in, as well as their religious attitudes [37,38].

In this study, the couples were also asked about their preferred sexual positions during pregnancy. The most commonly preferred sexual position was the knee–elbow position and on their side; a small subset of couples indicated having oral–genital contact, which was only recorded for the first trimester by men (fellatio). For the majority of religious women, only intergenital contact, i.e., penetration—is acceptable, with rare use of oral–genital stimulation or anal intercourse. Pregnant women with a more liberal approach to the principles of the Catholic Church have a different perspective; such women more commonly partake in general sexual contact and are characterized by greater freedom in choosing sexual positions and types of sexual stimulation [39].

Pioneering results were achieved by asking the study group about masturbation and erotic dreams during pregnancy. In this study, for men, the frequency of masturbatory behaviors occurring during pregnancy increased statistically significantly and was statistically significantly correlated with high values of sexual satisfaction. The increase in masturbation in men during pregnancy may be a natural occurrence and a solution to sexual tension [51].

In the literature, we read that men, as sexual beings from birth until death, engage in many behaviors throughout life to satisfy their sex drive [52,53,54]. Papp et al. [55] indicated the existence of a societal double standard regarding the masturbatory behavior of people (sexual double standard (SDS)). They highlight that, as a rule, women who masturbate are judged more harshly than men [56]. On the other hand, Haus et al. [57], based on research they conducted, noted that female masturbation was better perceived in society. Furthermore, women masturbated due to pleasure motives, but also due to intimacy reasons to a greater extent than men, highlighting that these motives help to explain the reverse SDS [58].

The Catholic Church teaches that masturbation is an immature act and brings about many negative effects [56]. Today, however, the vast majority of scientific publications on the matter of masturbation describe it not only as a natural function undertaken from an early age but also as a therapy used in treating premature ejaculation [59,60] or even a form of rehabilitation after myocardial infarction, to gradually return to full sexual performance [61].

The participants were asked about satisfying one’s own needs as well as the needs of partners, and most of the respondents, both men and women, stated that their relationship with their partner did not change at all during pregnancy. However, it should be noted that the study indicated that, for men, the percentage of those who did not have their sexual needs satisfied increased.

This may be a result of growing demands towards their partners and vice versa, in connection with increasingly accessible messages suggesting what “perfect” sex should look like or what a “perfect” or “ideal” partner should be. Another factor complicating successful intercourse may be a lack of dialogue, which may be due to a lack of knowledge regarding matters such as mutual feelings, sexual fantasies, or anxiety related to unwanted treatment in intimate situations [61]. The performed analysis indicated that, for women, the self-assessment score was compatible with the score obtained from the standardized questionnaires, where a low self-assessment accompanied a low score on the objectively assessed scale (the DSSS). The statistical analysis indicated that the average overall DSSS score of the examined women in the first trimester was statistically significantly higher than in the remaining two trimesters; furthermore, there was a statistically significant decrease in overall sexual satisfaction between the trimesters. The opposite situation was observed in men, with high self-assessment scores accompanying high scores in the assessment conducted using the sexual satisfaction scale (DSSS). It also indicated a correlation between DSSS and the frequency of sexual contact for women, for whom a low sexual satisfaction score accompanied a decrease in the frequency of sexual contact.

Of course, the study we conducted has a number of limitations. Therefore, further, in-depth analyses are necessary. Firstly, it was advisable to extend the analysis to include other standardized questionnaires, such as, for example, the Female Sexual Function Index or The International Index of Erectile Function Questionnaire. It would be important to conduct a larger population assessment, a multisite study, or a comparative study between populations. Another analysis could include not only the assessment of couples’ sexuality during the first pregnancy, but also subsequent pregnancies. This would make it possible to define sexual behavior according to the number of pregnancies. It is also worth considering not only heterosexual couples in the analysis, as well as extending the study to people whose biological sex is different from their psychological gender. It is possible that an incentive effect for participation in the study could be considered in a subsequent study. This would result in a greater diversity of participants from varying socioeconomic backgrounds. Despite the indicated limitations of our study, its results are significant. First, we confirmed that pregnancy that proceeds in a physiological manner is not a contraindication to sexual activity. Limitations, changes in the frequency of sexual activity, and its forms primarily depend on our views, behavior patterns, and our cultural and religious patterns that have been passed on to us. It has been shown that during pregnancy, both partners still have sexual needs that they want to satisfy. Again, however, perhaps because of the stereotypes, complexes, and cultural archetypes that exist, masturbation is more likely to be male than female. The results concerning the sources of obtaining knowledge about sexual activity during pregnancy seem disturbing. As we have shown, only 31% of women and 20% of men used professional counseling in this area. The largest number of respondents used sources such as the Internet or magazines. This may indicate that sexuality, including during pregnancy, is still a taboo subject in Poland; hence, reaching for sources that can be read anonymously in the comfort of your own home. Thus, this shows that in Poland there is a lack of sexual education, social campaigns concerning sexuality (including during pregnancy), trust in the health service and reliable sources of knowledge. A key factor may also be the fact that in the education of doctors and midwives, there is not enough emphasis on developing soft skills, including communication skills. Gynecologists and those caring for pregnant women should take the initiative on their own and talk to couples about changes in sexual activity during pregnancy. Therefore, in addition to local actions, systemic actions are necessary.

## 5. Conclusions

In both groups, the course of pregnancy may determine sexual dysfunction, which is noticeable mainly in the third trimester of pregnancy. Sexual satisfaction and frequency of intercourse decrease among couples during pregnancy. Pregnancy does not modify partnerships, but sexual problems during pregnancy may have a negative impact on the relationship and constitute additional stressors for couples. Medical staff should be trained in the assessment of sexual difficulties in people during pregnancy, in order to conduct reliable education and increase the awareness of couples regarding sexual and reproductive health.

## Figures and Tables

**Table 1:** Inclusion and exclusion criteria from this study.

Inclusion Criteria	Exclusion Criteria
Consent to participate in the study	Cack of consent for participation in the study
Heterosexual couple with a single physiological pregnancy	complicated pregnancy (at risk of preterm labor, diabetes, arterial hypertension, placenta previa, cervical insufficiency, in vitro pregnancy)
	Treatment or diagnosis of infertility
First-time pregnancy, diagnosed during the first trimester of pregnancy (up to the 12th week)	Treatment of diagnosis of sexual dysfunction
Regular gynecological visits during pregnancy, in line with the plan of care for the pregnant woman	Incorrectly completed or incomprehensible questionnaires
Correct completion of the set of research questionnaires by people in certain pregnancy trimesters.	Significant gaps in the responses to the questionnaires
	Insufficient knowledge of the Polish language in speech and writing

**Table 2:** Characteristics of the study group based on answers to questions in metrics.

Parameter		Women	Men	*p*-Value
Place of residence	Village	14	4	*p* = 0.25
	Urban < 100,000 citizens	68	86	*p* = 0.19
	Urban > 100,000 citizens	18	10	*p* = 0.43
Education	Vocational education	8	8	*p* = 1.0
	Secondary education	16	32	*p* = 0.10
	Higher education	76	60	*p* = 0.16
Occupation	Blue-collar worker	14	54	*p* = 0.27
	White-collar worker	76	40	*p* = 0.52
	Unemployment	10	6	*p* = 0.85
Relationship status	Marriage	66	66	*p* = 1.0
	Partnership	34	34	*p* = 1.0
Duration of relationship (years)		3.22 ± 0.23	3.80 ± 0.14	*p* = 0.02

*p*-value of the Student’s *t*-test; comparison between women and men for one parameter; mean ± standard deviation.

**Table 3:** Assessment of the relationship and physical attractiveness of the study group.

Factor	Women	Men	*p*-Value
Assessment of the relationship	8.64 ± 0.18	8.84 ± 0.21	*p* = 0.92
Assessment of own physical attractiveness	4.88 ± 1.37	7.2 ± 0.14	*p* = 0.00 *
Assessment of partner’s physical attractiveness	8.7 ± 0.16	9.4 ± 0.11	*p* = 0.76

* Statistically significant differences (Student’s *t*-test); mean ± standard deviation.

**Table 4:** Masturbation during pregnancy.

	First Trimester		Second Trimester		Third Semester		*p*-Value of the Post Hoc Tukey Test
	* *p* = 0.00		* *p* = 0.00		* *p* = 0.00		
Frequency	Women (*n* = 28)	Men (*n* = 76)	Women (*n* = 8)	Men (*n* = 100)	Women (*n* = 8)	Men (*n* = 100)	
1	0	20	0	4	0	8	*p* = 0.00 ^a,b^
2	0	30	0	40	0	32	*p* = 0.00 ^a,b^
3	0	26	0	56	0	60	*p* = 0.00 ^a,b^
4	28	0	2	0	2	0	*p* = 0.00 ^a,b^

1—once a week; 2—a few times a week; 3—daily; 4—less than once a week; *—statistically significant difference (Student’s *t*-test; *p* < 0.05); ^a^ Post hoc Tukey’s test for women (*p* < 0.05); ^b^ Post hoc Tukey’s test for men (*p* < 0.05).

**Table 5:** The sexual satisfaction self-assessment (SARSS) and sexual satisfaction scale (DSSS) during the course of pregnancy.

	First Trimester		Second Trimester		Third Semester		*p*-Value
	Women (*n* = 100)	Men (*n* = 100)	Women (*n* = 100)	Men (*n* = 100)	Women (*n* = 100)	Men (*n* = 100)	
Self-esteem	7.24	8.82	5.88	8.78	5.78	8.58	*p* = 0.05 * p = 0.99 **
Sexual satisfaction scale	71.02 ± 10.66	73.20 ± 10.72	64.28 ± 8.39	72.64 ± 12.15	63.08 ± 10.55	72.18 ± 10.55	*p* = 0.00 * p = 0.00 **

Mean ± standard deviation; * Post hoc Tukey’s test in women (*p* < 0.05); ** Post hoc Tukey’s test in men (*p* < 0.05).

## Data Availability

The data used to support the findings of this study are included in the article. The data will not be shared due to third-party rights and commercial confidentiality.
